# A Life-Threatening Urinary Emergency: Radiologic Characterization of Emphysematous Pyelonephritis

**DOI:** 10.1590/0037-8682-0011-2026

**Published:** 2026-06-15

**Authors:** Ramazan Kerim Yılmaz, Huseyin Er, Kemal Bugra Memis, Ayse Sena Celik, Sonay Aydin

**Affiliations:** 1 Mengucek Gazi Education and Research Hospital, Department of Radiology, Erzincan, Turkey.

**FIGURE 1:**
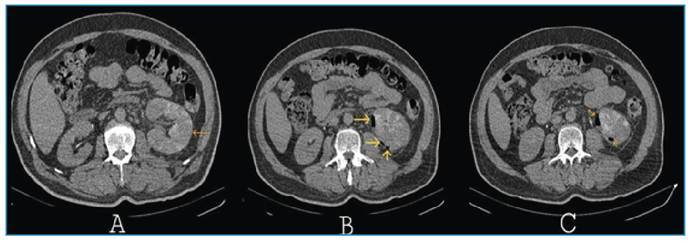
A: Computed tomography revealed a swollen area. B, C: Computed tomography revealed loculated gas collections in the lower left kidney.

A patient with diabetes mellitus and arterial hypertension presented to the emergency department with flank pain and fever. Computed tomography (CT) revealed a swollen area and loculated gas collections in the left lower kidney (Figure 1). Blood cultures revealed extended-spectrum beta-lactamase-positive *Escherichia coli*. Intravenous meropenem and metronidazole therapy was initiated. The patient's condition improved after two weeks of antibiotic therapy, and he was discharged with clinical recommendations.

Emphysematous pyelonephritis (EPN) is a severe kidney infection associated with high rates of renal loss and mortality. It is an acute necrotizing infection of the renal parenchyma and surrounding tissues. Gas may be present in the renal parenchyma, collecting system, or perinephric fat^1^. The clinical manifestations of EPN can be nonspecific; however, the clinical triad of fever, flank pain, and nausea is most common. Several classification methods for EPN exist for both plain radiographs and CT. Wan et al. used CT to define Type I (necrosis with gas but no fluid collection; worse prognosis) and Type II (parenchymal gas with fluid collections; better prognosis)^2^. Diabetes mellitus is the most significant risk factor, occurring in 90% of EPN patients^3^. EPN treatment options have evolved over time, ranging from aggressive operations to more conservative approaches consisting primarily of drainage and medication^4^.
